# Psychoanalytical Considerations of Emotion Regulation Disorders in Multiple Complex-Traumatized Children—A Study Protocol of the Prospective Study MuKi

**DOI:** 10.3389/fnhum.2022.809616

**Published:** 2022-04-26

**Authors:** Felicitas Hug, Tom Degen, Patrick Meurs, Tamara Fischmann

**Affiliations:** ^1^Sigmund-Freud-Insitut, Frankfurt, Germany; ^2^Clinical Psychology, Faculteit Psychologie en Pedagogische Wetenschappen, KU Leuven, Leuven, Belgium; ^3^Neuropsychoanalyse, Klinische Psychologie, Psychotherapie und Psychoanalyse, Internationale Psychoanalytische Universität, Berlin, Germany

**Keywords:** multiple complex traumatization, emotion regulation, neurobiological and behavioral studies, reward expectancy, attachment behavior, young children (8–12 years)

## Abstract

Studies in adults with mental disorders suggest that the experience of early and chronic trauma is associated with changes in reward expectancy and processing. In addition, severe childhood trauma has been shown to contribute to the development of mental disorders in general. Data on effects of early childhood trauma on reward expectancy and processing in middle childhood currently appear insufficient. The present study aims to fill this research gap by examining the effects of developmental trauma disorder (DTD) on reward expectancy and processing in children aged 8–12 years, testing the hypothesis that children with multiple complex traumas exhibit altered reward processing as a result of prior disappointing reward experiences. One main feature of developmental trauma disorder is early experiences of multiple separation from important and close relationships alongside other experiences of emotional or physical harm. In the sequel children often show affect regulation disorders. To investigate this, we have developed an adapted version of the Monetary Incentive Delay (MID) Task, which examines children’s expectation of reward or frustration. In this first study, behavioral data will be collected from *N* = 40 children (*n* = 20 experimental group and *n* = 20 healthy controls) using this adapted version of the MID Task. Children in the experimental group will be recruited from youth welfare centers in Frankfurt a.M., Germany. Healthy control subjects will be recruited from after-school-care facilities. A brief trauma screening will be conducted for both groups, experimental and control. If children show signs of trauma, the presence of a developmental trauma disorder will be further delineated by a German translation of the Developmental Trauma Disorder Structured Interview for Children (DTDSI-C) which was translated the first time in German by our research group. We hypothesize that children in the experimental group will be less accurate in performing the Monetary Incentive Delay Task because of their impaired emotion regulation skills due to emotional avoidance following developmental trauma. If the results of our initial behavioral study are promising, the MID task will be used in a future study to elucidate the relationship between trauma developmental disorder, reward expectancy and processing, and neurobiological processes in middle childhood.

## Introduction

Multiple complex-traumatized children frequently fall through the cracks in our health care system and are often hospitalized with different treatment approaches for different diagnoses due to a lack of a comprehensive understanding of their emotional experience and its processing. It is difficult for these children to come to peace, which is a prerequisite for more positive psychosocial development. The movie “Systemsprenger”^1^ impressively shows the difficulties multiple complex-traumatized children have in learning and how threatening benevolent relationship attempts have on them against the background of their aversive previous relationship experiences. Very early, unprocessed trauma, common in multiple complex-traumatized children, may continue to have an impact in subsequent developmental stages as “cumulative trauma” (Khan, 1963). Developing children are particularly vulnerable after severe trauma. Thus, children’s fantasies about the unprocessed trauma, expressed in fears or futile explanation attempts, can even lead to increasing vulnerability (Greenacre, 1967). Therefore, dealing with trauma in a holding and secure relationship after traumatization is crucial to avoid re-traumatization, pathological adjustment, or developmental arrest (Keilson, 2007). The sensitization and accompaniment of parents, social workers and foster parents is equally important for trauma-sensitive care of affected children. However, the existing offers of help from youth welfare offices and pediatricians are aimed at already acquired socio-cognitive skills as well as the acquisition of new skills. However, if basic learning processes are disturbed, it is necessary to develop offers that initially promote a positive expectation in a trauma-sensitive manner to make learning possible in the first place (Zimmermann, 2017). According to Tops et al. (2014), the ability to form close and supportive relationships also depends on specific neurobiological processes (see “Excursus and Following” section). Considering this aspect relationship experiences are also shaped neurobiologically in addition to an innate need for attachment (Bowlby, 1958). Neurobiological processes influence motivation, learning and emotion regulation on the behavioral level. According to Tops et al. (2014), during the development of very young children, there is a shift from an initial turning to the first attachment figures as completely new relationships, usually the parents, to seeking already familiar relationships, usually also the parents. This switch is closely linked to neurochemical processes in the brain’s reward system and is associated with different neurotransmitters and distinct processing pathways (see “Excursus and Following” section). With this shift, “internal working models” (Bretherton and Munholland, 2016) regarding which relationship expectations exist and are experienced as rewarding are internalized. At the neurobiological level, this switch occurs from ventral corticolimbic pathways to dorsal ones. While ventral pathways are associated with fast, associative learning, dorsal pathways are associated with internal, slower processing modalities and lower emotional reactivity. Mesolimbic dopamine areas contribute to the shift from one processing modality to the other, where the bonding hormone oxytocin plays a mediating role, favoring the formation of familiar relationships. That is, early in development, newborns seek relationships with their first, new attachment figures, and only after the neurobiological shift from ventral to dorsal processing will they associate familiar relationship patterns with reward. Thus, preference for familiar and trusted relationship patterns will emerge, whereas new relationship experiences may then be more reluctantly pursued, neurobiologically even prevented, and not further reinforced (Tops et al., 2014). This neurobiological background is presumably responsible for the fact that people find it so difficult to break away from early, dysfunctional, but familiar, relationships, or also why children nevertheless protect the relationship with their parents who harm them. Secure inner working models cannot be formed or can only be partially formed due to dysfunctional, often inconsistent, and unreliable parental responses to the infant. Chaotic and unpredictable family circumstances, from which multiple complex-traumatized children often originate, consequently lead a child to perceive his social environment and thus also relationship offers as unpredictable and threatening; the child has generalized its internal working models for relationships. This leads to an increase in emotional arousal, affective tension, and emotional reactivity in social situations. The unpredictability of the social environment is thus also associated with attachment insecurity.

## Materials and Methods

The project “Mutige Kinder—MuKi” (courageous children) is a venture for basic scientific research into the above-described developmental risks in the socio-emotional area with the aim of investigating deviating developmental trajectories based on emotional processes and their effects on reward expectation and -evaluation processes and thus on individual development of cognitive skills and academic success. Prospectively, these findings can be incorporated into the development of early prevention measures. The object of the project “MuKi” is to research multiple complex-traumatized children who show deficits in their processing of emotions due to their psychosocial development. Children with multiple complex traumatization are characterized by frequent relationship disruptions with primary caregivers and early aversive experiences. They can become emotionally derailed in everyday life-situations, mistakenly evaluating those situations as existentially threatening. Children who are multiple complex-traumatized then often show (auto-) aggressive behavior or withdraw from social settings (Weinberg, 2010) in order not to avoid situations that emotionally overwhelm them and which they cannot regulate (Weinberg, 2010, p. 37). It can be assumed that children with multiple complex traumata show increased expectancy-anxiety and emotional avoidance in social situations, which ultimately affects their psychosocial and emotional experience and behaviors. In child and adolescent psychotherapies, it is often observed that the expectation of a reward cannot be experienced as something positive by severely traumatized children because they expect something negative to follow the reward due to their negative prior experiences. Consequently, their expectations are negatively distorted in a specific way. Reward does not put these children in a state of joyful expectation, but in a state of alarm. This specifically altered reward processing of multiple complex-traumatized children is to be investigated in the project “MuKi.”

### Excursus: Neurobiology of Attachment Relationships

Brain systems involved in the formation of attachment between parents and children are associated with mesolimbic processing pathways where dopamine (DA) is released in a quasi-rewarding manner (Cooper and Breese, 1975; Hernandez and Hoebel, 1988; Damsma et al., 1992; Yoshida et al., 1992; Young et al., 1992). The mesolimbic system is also associated with, among other things, motivational processes and the importance of reward incentives (Mogenson, 1987; Blackburn et al., 1992; Ikemoto and Panksepp, 1999; Wise, 2004; Berridge, 2007). Of relevance to our research is that activation of two classes of DA receptors in striatal regions are usually balanced (MacRae et al., 1987; LaHoste et al., 1993; Gerfen et al., 1995; Carlezon et al., 1996; Hu and White, 1997). When an imbalance occurs in favor of activation of either DA receptor, dependency disorders or impulse control disorders may result (Self et al., 1996; Perez et al., 2011; Grieder et al., 2012), which are common in multiple complex-traumatized children. According to Herman and Panksepp (1978), opioid receptors in the mesolimbic system as well as the associated nucleus accumbens (NAC) mediate social reward and motivation. The opioid system, in turn, interacts with the DA and oxytocin (OT) systems to generate a feedback loop to the DA and opioid receptors so that behavior and expectations are positively reinforced (Young and Alexander, 2012). Applied to relationships, this can be thought of as an arising initial euphoria in a new relationship which then subsides and is gradually replaced by a sense of satisfaction. This initial change can be understood as a development of tolerance, to which the release of opioids contributes. Consequently, cravings for the person increases and he or she is sought out more frequently, even if the person himself or herself evokes negative emotions or consequences (Burkett and Young, 2012). According to Burkett and Young, such neurobiological dependence can also occur in destructive relationships. Related to the parent-child relationship, this dependency can also be understood as a neurobiological predisposition that operates even when a child is neglected or even abused by the same parent. In relation to multiple complex-traumatized children and against the background of their negative relationship experiences, this means that negative expectations in social situations may be predisposed not only psychologically but also neurobiologically.

### Excursus: Incentive Salience and Reward Expectancy From a Neurobiological Perspective

Another concept, namely that of incentive salience, is also important for our research in question. The concept of incentive salience divides the brain’s reward system into two distinct processes, “Wanting” and “Liking,” with distinct neurobiological processing pathways that can lead to different motivations for reward. “Wanting” is psychologically and neurobiologically distinct from “Liking.” Furthermore, “Wanting” cannot be equated with wanting in the common sense, where the latter describes a desire in terms of “wanting to have,” which presupposes higher cognitive processing procedures in which links already exist about how a desire can be fulfilled. According to the concept of incentive salience, “Wanting” is generated by a neurochemical process on mesolimbic processing pathways in the brain that represents specific incentives that have Pavlovian properties related to reward. Accordingly, reward in the true sense cannot occur without incentive salience. “Liking” which is oriented toward pleasure is in itself a spontaneous affective state that does not require a desire for a reward. However, “Wanting” and “Liking” are jointly necessary for the sensation of a reward. The process of incentive salience attribution first leads to an incentive arousing desire (Berridge and Robinson, 2016). “Incentive salience wanting” is based on neurochemical processing at the subcortical level in the brain, where processes are automatic and unconscious, whereas more cognitive forms of “wanting to have,” as described above, occur at the cortical level, and are thus closer to consciousness. Crucially, here, “Wanting” can also lead to irrational desires that are neither wanted nor liked intellectually, as has been shown in addictive disorders (Berridge and Robinson, 2016). “Wanting” and “Liking” are thus associated with distinct motivational processes and regions in the brain that can be measured under fMRI conditions using established computerized testing procedures. “Wanting” is associated with reward expectancy, which we will investigate in our research project, hypothesizing that multiple complex-traumatized children will have lower reward expectancy than non-complex-traumatized children due to their negative prior experiences. Aversive relational experiences are what is familiar to multiple complex-traumatized children, which is why they do not expect anything good in social situations, even when someone approaches them with good intentions. It is to be expected that children from a developmentally stable environment, show more positive expectation toward others in social situations than multiple complex-traumatized children. Reward expectancy also affects children’s psycho-social development, and thus their ability to establish positive and supportive relationships, which is essential for positive development in life, and particularly relevant from a psychoanalytic perspective. The different reward expectations of multiple complex-traumatized children and a non-complex-traumatized comparison group will be investigated in the research project “MuKi.”

### The Neurobiology of the Reward System and Its Impact on Behavioral Decisions

Distinct neurobiological and learning systems underlie decision making and choice (Dolan and Dayan, 2013). Both systems control behavior depending on its consequences with the goal of obtaining reward and avoiding punishment (Dayan et al., 2006; Dayan and Daw, 2008). Humans show surprising inflexibility in making decisions where they must suppress behavior to obtain reward and can avoid punishment by exhibiting a certain behavior. This inflexibility can be explained by the fact that stimuli that are expected to produce a reward are intrinsically coupled with approach behavior, and those that leads to expectation of punishment are coupled with avoidance behavior. This response behavior to positive or aversive incentive stimuli, which is biased by its affective valence, is called “Pavlovian bias” and is considered to interfere with the flexible instrumental decision process (Cavanagh et al., 2013; Guitart-Masip et al., 2014).

From a neurobiological point of view—taking into account the behavioral systems of instrumental learning (actionality) and the “Pavlovian bias” (valence)—the reward system can be divided into an appetitive incentive component—“Wanting”—and an affective component—“Liking” (see above; Berridge and Robinson, 2016). In early development, the “Wanting System,” which is characterized by rapid associative learning (Tops et al., 2010, 2013), is more active in terms of exploration or curiosity. Then, when the infant begins to respond to and is receptive to the presence of attachment figures, there is a switch from processing via ventral limbic pathways (“Wanting”), to dorsal striatal control pathways (“Liking”). According to attachment research, this shift is crucial for the emergence of “Internal Working Models”—IWM (Bretherton and Munholland, 2008). In cases of neglect or maltreatment early in development, this switch is impeded, and appropriate IWMs cannot be formed (Tops et al., 2014). Furthermore, the decision to engage in goal-directed behavior (actionality) is influenced by the affective valence of preconceived expectations about the consequences of an action. As a result of traumatic experiences, humans and animals show difficulties in counteracting or resolving expectancy anxiety and avoidance behaviors (Milad et al., 2009; Jovanovic et al., 2010). This can lead to a permanent conditioned fear response and avoidance behavior because of a Pavlovian association chain and thus disrupt instrumental learning. In relation to the current study project, it can be assumed that negative expectations, expectancy anxiety, and emotion avoidance because of early traumatization and aversive attachment experiences influence reward processing as well as decision-making behavior in such a way that positive experiences are experienced as less positive and are less frequently sought out. In addition, due to a lack of positive experiences, aversive relationship experiences are interpreted as rewarding in the context of the attachment process. Therefore, the reward system also plays a prominent role in understanding these early attachment processes (Tops et al., 2014). The aim of our research project “MuKi” is to gain a better understanding of the processing of reward expectation and evaluation and its impact on behavior in multiple complex-traumatized children, which to our knowledge has not been investigated so far.

Furthermore, the underlying psychological processes and their neuropsychological correlates of behavioral problems that are very common in children following trauma, have not been adequately researched. Studies of adults with mental disorders have shown that traumatic experiences are associated with altered expectancy and appraisal processes (Cella et al., 2010; Sebold et al., 2014), which contributed significantly to the development of mental disorders (Huys et al., 2015). To examine how appraisal processes are psychologically and neurobiologically altered following early and chronic trauma in children is the goal of this research project. This should lead to a better understanding of the genesis of psychological developmental disorders. This understanding is also essential for the development of specific prevention services for multiple complex-traumatized children.

### Research Instruments

As the goal of this project is to explore reward expectancy and its effects on traumatized children’s experience and behavior, an experimental paradigm was programmed and adapted to our research question and specific sample, based on the MID Task (Knutson et al., 2000; Demurie et al., 2013). In this child-directed computer game, children can win different amounts of money on each task with measurements of how much the monetary incentive improves attentional performance. Intermittently, negative feedback is also provided regardless of the child’s performance, and no money can be won. This is to investigate how strongly and how persistently this demotivating feedback is reflected in the child’s performance. After the assessment, we take time to fully explain the deception and our intention behind it to the child and are available for open questions to relieve the child of the frustration that has arisen.

By using the MID Task paradigm, which is specifically designed to study the reward system, we are targeting behavioral and neural correlates of anticipation as well as of hedonistic processing of rewards (in terms of actionality vs. valence or “Wanting” vs. “Liking”), and of anticipation and processing of frustration. From a neurobiological perspective the MID Task first activates the ventral striatum in proportion to an expected gain. Subsequently, the valence (positive/negative) of the reward received activates parts of the ventro-medial frontal cortex (Knutson et al., 2000; Simon et al., 2010). This is specifically important to our project as previous studies (Mehta et al., 2010) demonstrated that orphans with early deprivation experience showed reduced activation of the ventral striatum in the anticipation phase indicating a lack of positive reward expectancy. Specific emotional processing (emotion avoidance because of traumatization) will thus be examined in the study using this paradigm to capture anticipated reward as well as in relation to attachment behavior, leading to a more comprehensive understanding of socio-emotional development in multiple complex-traumatized children.

In a first step, we intend to determine the transferability and sustainability of this paradigm by examining how the test situation maps to the behavioral level. If the paradigm proves to be effective in investigating reward expectancy and its effect on feelings of success and frustration during the test situation, this further developed paradigm will be used under fMRI conditions in a subsequent study.

In addition to the adapted MID task (Knutson et al., 2000), the focus of the current study “MuKi” is on the use of an interview on the child’s traumatic encounters and experiences with its family and separations from family members to investigate the occurrence of multiple complex traumatization. For this purpose, we were the first to translate the Developmental Trauma Disorder Structured Interview for Child (DTDSI-C) developed in the United States by Ford (Ford et al., 2018) into German and trained our staff in the specific implementation of the interview as well as in trauma-sensitive interaction with children. Before administering the DTDSI-C we ensure the presence of trauma via a brief trauma screening, using part of the Diagnostic interview for mental disorders in childhood and adolescence (Diagnostisches Interview bei psychischen Störungen im Kindes- und Jugendalter—K-DIPS; Schneider et al., 2017). In addition, standardized psychological testing procedures are used to control for bias due to associated psychological concepts such as attention bias (d2-R; Brickenkamp et al., 2010) or basic school prerequisite knowledge, language comprehension, and intelligence (AID-2; Kubinger and Wurst, 2000).

### Participants and Procedure

Since the manifestation of multiple complex traumatization is to be expected especially in children who had to be taken into care and are subsequently in foster families or institutions, a cooperation with the youth welfare center in Frankfurt a.M., Germany was established. The children to be examined are either placed directly by the youth welfare center or by family support agencies commissioned by the youth welfare center. From an ethical perspective, participation in the study for children with early trauma is only appropriate if there is currently continuity and stability in the relationship with a caring professional. Exclusion criteria for participation in the experimental group are the use of psychotropic drugs or other drugs that influence the perception of emotions, such as antidepressants or anxiolytics. Inclusion criteria are at least one separation experience from significant caregivers and the presence of multiple complex traumatization. After a suspension due to the Corona pandemic, data collection is currently taking place again, provided that the infection levels allow it and that the family service organizations cooperating with us continue to maintain their regular operations. The study is aimed at children aged 8–9 years. The youth welfare center of the City of Frankfurt a.M. has received information packages for interested families through the Sigmund Freud Institute. Families or foster parents interested in the study can give their consent to be contacted by our study staff. A pool of children interested in taking part in the control group exists from previous projects. Children undergoing therapy or taking psychotropic drugs will be excluded from participation in the control group. In addition, children are only included in the control group if they were inconspicuous in the K-DIPS. If the children lack German language skills, they cannot participate in either of the two examination groups, as we have to make sure that the children understand our instructions, which is why we generally also check language comprehension (AID-2). In all cases, the parents are contacted by telephone and a brief initial explanation is given about the background, the process, and the aim of the “MuKi” project. If there is continued interest in participating in the study, children accompanied by a parent or caregiver will be invited to an initial appointment at our center. The first appointment includes an initial assessment of the child by an experienced child and adolescent psychotherapist, who assesses the child’s ability to cope with the study situation using the Children’s Global Assessment Scale (CGAS; Schaffer et al., 1983). Children for whom study participation might be too upsetting or potentially retraumatizing can be identified and excluded early on through this process, so that our study does not adversely affect the child.

For third-party assessment of the children, caregivers or foster parents are interviewed with the Teacher Report Form (TRF; Döpfner et al., 2014) questionnaire to obtain a comprehensive impression of the children and their socioemotional difficulties and to learn about their special needs. The children are accompanied by trauma-sensitive trained staff or the caregiver during the entire assessment, which takes place over three appointments. If children have a particularly extensive need for discussion, an additional fourth appointment is arranged so that the total duration of the respective appointments can always be arranged in a child-friendly scope. All children receive an expense allowance in the form of a cinema or toy voucher of 20€ for participating in the “MuKi-Study.” If necessary, referral to outpatient psychoanalytic or depth psychology-based child psychotherapy is made. A parent consultation, in which the clinical observations are summarized and gently conveyed by the study director, is also offered as an option. Taking into consideration the behavioral measures of the MID-task (Error rate, response time) comparing means of 2 independent equal sized groups (assuming normal distributions and homogeneous variances), with a power of 0.80 and a Type 1 error rate of 0.05 a sample size of *N* = 40 children (*n* = 20 experimental group multiple complex-traumatized children; *n* = 20 non-traumatized control group) was calculated to detect group differences. All appointments will be conducted in compliance with standard Corona protective measures and after having undergone the review of the ethic commission of the IDeA Centre^2^. Ethical approval has been obtained from the DIPF ethical committee (DIPF—Leibniz-Institut für Bildungsforschung 16.04.2021).

## Discussion

The project “MuKi” has a high societal relevance as its goal is to better identify and deal with multiple complex-traumatized children in the future. In our view, the conditions for these children will continue to worsen. This is evident from the fact that already during the first lockdown during the Corona pandemic, significantly fewer children were taken into care, although the need for this increased (cf. HR podcast of 28.04.2020). An increased need with fewer custody case slots speaks primarily to the fact that many children have to endure adverse circumstances for a longer period of time and are therefore more likely to develop more severe emotional problems, which in turn require a more precise professional understanding. Only if these problems are recognized in a differentiated way prevention programs can be targeted in the future. Children with multiple complex-traumatization are often taken into care only after chronic and repeated trauma, and only after the children concerned have exhibited multiple behavioral problems. In most cases, these children are already stigmatized in the school context or other social contexts and have undergone various psychiatric and behavior modification measures. It often takes years for multiple complex-traumatized children to receive appropriate care and therapeutic support, during which re-traumatization may occur and behavior problems may be manifested. The various diagnoses and treatments given in different institutions in health care and urban, social institutions for these multiple complex-traumatized children show that a comprehensive understanding of the consequences of early and complex traumatization is still missing, especially in the area of emotion regulation and reward expectation, which precede behavioral decisions made by these children. In order for these children to be diagnosed at an earlier stage and to save them the long path through various treatments, the “MuKi” project is dedicated to basic research in the area of emotion regulation disorders and reward expectations in multiple complex-traumatized children. The Corona pandemic, in which children in particular suffered from a lack of social and educational opportunities outside the family, has perhaps made the need for such research even clearer.

The current project is a first study in a series of studies whose goal is to ultimately gain in-depth knowledge not only about the psychological and socio-emotional but also on the specific neurobiological development of multiple complex-traumatized children. We consider this to be important because we assume that early and chronically traumatized children differ in their development from non-traumatized children and are on a different developmental level than children who grow up carefree. The experience of a severe trauma cannot only lead to an arrest of development in some areas, so that an adolescent may still behave in a very childlike manner, but also neurobiological maldevelopments can occur in certain brain areas very early on. In addition to cognitive structures, the experience and expression of emotions and the ability to form relationships are often affected. The knowledge gained from the current project will help to more comprehensively identify the special needs of early traumatized children also from a neurobiological point of view in a subsequent fMRI study and, supported by scientific research, can be incorporated into the development of needs-based prevention services. Considering the possible arrest of psychological and neurobiological development in early multiple-complex traumatized children, their individually needed treatment must be adapted to their specific stage of arrested development. Existing interventions based more on behavioral and/or cognitive approaches may overwhelm such a child, and his or her low response to treatment may then be misinterpreted as refusal, developmental delay or lack of intelligence. Research into basic scientific aspects of emotion regulation disorders and reward expectancy in children with multiple complex traumas and the development of need-based prevention services can be carried out within the framework of existing support systems (youth welfare office, family services). Prevention can thus be underpinned with basic scientific research and adapted to the changing, specific needs of children and adolescents in the best possible way and made as accessible as possible. In addition, by generating psychological and neurobiological data that can be obtained through a subsequent fMRI study, a stronger negotiating position with funding agencies, such as statutory health insurance or municipal funding agencies, can be achieved.

A first goal that we have already been able to achieve within the framework of the present project is the good and continuous cooperation with the decision-makers of the youth welfare office of the City of Frankfurt a.M. Limitingly, given the high needs of this group of children, a well thought-out and designed project can always fail in practical implementation if the families and children do not accept the programs or if their access is hindered by language and bureaucratic hurdles. Through the described cooperation with the youth welfare center, this hurdle has already been overcome to a large extent.

Further limitations that need to be mentioned here are the relatively wide age range of children aged 8–12 and the associated developmental differences that may present hurdles in data evaluation and interpretation. These developmental differences may affect cognitive abilities such as semantic comprehension or numerical reasoning and thereby affect the monetary incentive, which is particularly relevant in the present project due to the use of the adapted MID task that examines reward expectancy. Furthermore, developmental differences may also be evident in emotional maturity, symbolizing and mentalizing abilities (Fonagy et al., 2002). In later studies, the age range may be narrowed even further. The age range was initially chosen so generously in order to be able to include as many children as possible from this sample. Other limitations that need consideration are developmental regression or consolidation in latency (Pestalozzi, 2014), which may also complicate interpretation of results. In all cases, data interpretation should be cautious, and assumptions can only be made by formulating hypotheses and looking at correlations for the time being. However, this study should also be viewed as the first study in a series designed to gather initial data and experience regarding the sample, the instruments, and not least the accessibility of the sample.

Finally, we would like to emphasize that multiple complex traumatization should be considered as a multimodal developmental disorder (Weinberg, 2010) in which child development is comprehensively affected. Therefore, it is difficult to examine and consider only one developmental area in isolation. Since many areas of child development are affected by early and chronic trauma, there is also much overlap to adjacent and delineated developmental concepts. As described in the “Materials and Methods” section, we control for some of these concepts, such as attention bias (d2-R) or basic school knowledge, language comprehension, and intelligence (AID-2). However, even if we control for these associated psychological concepts, a clear interpretation of the results remains difficult, as emotion avoidance and a negative reward expectancy can block positive developmental and learning processes. Because of the multimodality of early and complex trauma, studying its consequences is very costly and time-consuming, which is reflected in our extensive study design. In our view, however, such an elaborate study design is necessary to gain a comprehensive understanding of deviant developmental trajectories because of chronic traumatization.

## Ethics Statement

The studies involving human participants were reviewed and approved by the Ethics Committee of the DIPF—Leibniz-Institut für Bildungsforschung und Bildungsinformation. Written informed consent to participate in this study was provided by the participants’ legal guardian/next of kin.

## Author Contributions

PM and TF conceptualized the topic. FH and TF systematically reviewed the literature, drafted the first draft of the manuscript, and were jointly accountable for the content of the work, ensuring that all aspects related to accuracy or integrity of the study are investigated and resolved in an appropriate way. TD provided experimental expertise in developing instruments for the project and has written these parts of the article. All authors contributed to the article and approved the submitted version.

## Conflict of Interest

The authors declare that the research was conducted in the absence of any commercial or financial relationships that could be construed as a potential conflict of interest.

## Publisher’s Note

All claims expressed in this article are solely those of the authors and do not necessarily represent those of their affiliated organizations, or those of the publisher, the editors and the reviewers. Any product that may be evaluated in this article, or claim that may be made by its manufacturer, is not guaranteed or endorsed by the publisher.

## Footnotes

1
https://www.systemsprenger-film.de/

2
https://www.idea-frankfurt.eu/en/about-idea
